# Validation of antibiotic stewardship metrics for genitourinary infection management in Veterans Affairs outpatient settings

**DOI:** 10.1017/ash.2023.264

**Published:** 2023-09-29

**Authors:** Jordan Braunfeld, Matthew Samore, Jacob Crook, McKenna Nevers, Kelly Echevarria, Ben Brintz, Matthew Goetz, Karl Madaras-Kelly

## Abstract

**Background:** Diagnosis and management of suspected urinary tract infection (UTI) in outpatient settings has been shown to be suboptimal. We previously developed a set of stewardship metrics for UTIs based on electronic health record (EHR) data (*Antimicrobial Stewardship & Healthcare Epidemiology* 2022;2 suppl 1:S5–S6. doi:10.1017/ash.2022). A tier-based approach was used to more fully capture antibiotic use associated with genitourinary (GU) symptoms and diagnoses. Herein we report a preliminary analysis of validity and reliability of these metrics based on chart abstraction. **Methods:** The study cohort consisted of patients who visited Veterans Affairs emergency departments or primary care clinics between 2015 and 2022 and who had a GU diagnosis based on *International Classification of Disease, Tenth Revision* (ICD-10) codes, divided into 3 categories: tier 1 (antibiotics always indicated), tier 2 (antibiotics sometimes indicated), and tier 3 (antibiotics not indicated). Visits related to urological procedures, nontarget settings, or concomitant non-GU infections were excluded. Cases were randomly sampled for manual review from within 8 strata based on tier, use of antibiotics, and visit type. An infectious disease physician and pharmacist abstracted charts using a standardized data-collection instrument. Clinical judgments regarding diagnosis and treatment were recorded on a Likert scale without knowledge of how the patient was managed. The intraclass correlation coefficient (ICC) was used to estimate interrater reliability. **Results:** To date, 148 cases have been reviewed (50 by both reviewers). Mean (SD) age was 67.5 (15.3) years and 12.2% were female. In a majority of tier 1 and 2 visits in which antibiotics were given, the reviewers found evidence for GU infection (69.7%) and favored prescribing of antibiotics (60.6%) (Table). In contrast, most patients in the tier 3 category who received antibiotics were judged to have noninfectious conditions (eg, benign prostatic hypertrophy) and to not require antibiotics. In the subset of records examined by both reviewers, the interrater reliability of judgments of whether antibiotics were warranted was good (ICC = .704). **Conclusions:** This preliminary validation provides support for a tier-based approach for stewardship metrics for GU conditions that relies upon electronic data to identify patients for whom antibiotics are generally not indicated.

**Figure:**
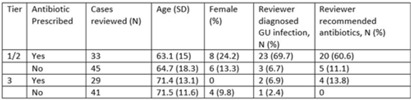

**Disclosures:** None

